# mStrain: strain-level identification of *Yersinia pestis* using metagenomic data

**DOI:** 10.1093/bioadv/vbad115

**Published:** 2023-09-15

**Authors:** Xiuwei Qian, Yarong Wu, Xiujuan Zuo, Xin Peng, Yan Guo, Ruifu Yang, Xianglilan Zhang, Yujun Cui

**Affiliations:** School of Basic Medical Sciences, Anhui Medical University, Hefei 230032, China; State Key Laboratory of Pathogen and Biosecurity, Beijing Institute of Microbiology and Epidemiology, Beijing 100071, China; State Key Laboratory of Pathogen and Biosecurity, Beijing Institute of Microbiology and Epidemiology, Beijing 100071, China; School of Basic Medical Sciences, Anhui Medical University, Hefei 230032, China; State Key Laboratory of Pathogen and Biosecurity, Beijing Institute of Microbiology and Epidemiology, Beijing 100071, China; State Key Laboratory of Pathogen and Biosecurity, Beijing Institute of Microbiology and Epidemiology, Beijing 100071, China; State Key Laboratory of Pathogen and Biosecurity, Beijing Institute of Microbiology and Epidemiology, Beijing 100071, China; State Key Laboratory of Pathogen and Biosecurity, Beijing Institute of Microbiology and Epidemiology, Beijing 100071, China; School of Basic Medical Sciences, Anhui Medical University, Hefei 230032, China; State Key Laboratory of Pathogen and Biosecurity, Beijing Institute of Microbiology and Epidemiology, Beijing 100071, China; School of Basic Medical Sciences, Anhui Medical University, Hefei 230032, China; State Key Laboratory of Pathogen and Biosecurity, Beijing Institute of Microbiology and Epidemiology, Beijing 100071, China

## Abstract

**Motivation:**

High-resolution target pathogen detection using metagenomic sequencing data represents a major challenge due to the low concentration of target pathogens in samples. We introduced mStrain, a novel *Yesinia pestis* strain/lineage-level identification tool that utilizes metagenomic data. mStrain successfully identified *Y. pestis* at the strain/lineage level by extracting sufficient information regarding single-nucleotide polymorphisms (SNPs), which can therefore be an effective tool for identification and source tracking of *Y. pestis* based on metagenomic data during a plague outbreak.

**Definition:**

**Strain-level identification:**

Assigning the reads in the metagenomic sequencing data to an exactly known or most closely representative *Y. pestis* strain.

**Lineage-level identification:**

Assigning the reads in the metagenomic sequencing data to a specific lineage on the phylogenetic tree.

**canoSNPs:**

The unique and typical SNPs present in all representative strains.

**Ancestor/derived state:**

An SNP is defined as the ancestor state when consistent with the allele of *Yersinia pseudotuberculosis* strain IP32953; otherwise, the SNP is defined as the derived state.

**Availability and implementation:**

The code for running mStrain, the test dataset, and instructions for running the code can be found at the following GitHub repository: https://github.com/xwqian1123/mStrain.

## 1 Introduction

Accurate strain-level target pathogen identification is critical for public health surveillance, especially for controlling disease outbreaks (Buytaers *et al.* 2021). Metagenomic sequencing is an efficient and advanced technology for the study of microbiomes and target pathogen detection (Quince *et al.* 2017). The accurate identification of target pathogens in metagenomic data is of major importance. However, the low abundance of target pathogens in a sample complicates their identification. Most state-of-the-art bioinformatics tools, such as MetaPhlAn2 (Truong *et al.* 2015) and GTDB-Tk (Chaumeil *et al.* 2019), can only identify pathogens in metagenomic data at the genus or species level (Anyansi *et al.* 2020). To this end, these tools align sequencing reads against reference genomes of the designated species (Li *et al.* 2015, Truong *et al.* 2017, Asnicar *et al.* 2020). Then the sequence similarity or phylogenetic relationships are analyzed to identify the specific target pathogen (Luo *et al.* 2015, Wang *et al.* 2023).

Nevertheless, the sequence alignment methods mentioned above depend on broad databases encompassing numerous species. Such a universal sequence alignment strategy for pinpointing target pathogens at the strain level comes with inherent limitations. First, we need to manually set a sequence similarity cutoff threshold largely based on experience, which is difficult for novice bioinformaticians. Second, we can hardly acquire sequence assemblies due to the low abundance of target pathogens in the test sample. On the other hand, it is common for the presence of highly conserved genomic regions that are shared among different species. These “interspecies repeats” combined with the low coverage of most species, may trigger intergenomic assembly errors (Nurk *et al.* 2017). Third, the task of aligning raw reads to the comprehensive genome dataset, encompassing reference genomes of multiple species, proves to be a time-intensive endeavor.

*Yersinia pestis* is the pathogen responsible for plague outbreaks and has caused three pandemics in history (Barbieri *et al.* 2021). If not treated promptly, *Y. pestis* infection can cause life-threatening complications. Accurate identification of *Y. pestis* in metagenomic data is critical for monitoring plague threat. By accurately identifying the strain type of *Y. pestis*, we can gain valuable insights into the origin of the *Y. pestis* infection. This knowledge serves as a crucial foundation for clinical treatment, enabling healthcare professionals to tailor their approaches to effectively combat the specific strain involved.

For precise and rapid identification of strain-level target pathogens within metagenomic data, we introduce mStrain (strain-level identification in metagenomic data). In contrast to depending on a comprehensive sequence database that spans multiple species, mStrain focuses solely on the single-nucleotide polymorphism (SNP) loci found within the genomes of the specific target pathogens. These SNPs effectively capture the distinctions among different *Y. pestis* strains. In this context, we utilize the mStrain methodology to successfully attain strain-level identification of *Y. pestis* through metagenomic data.

## 2 Methods

### 2.1 Framework of mStrain

**Figure 1.**
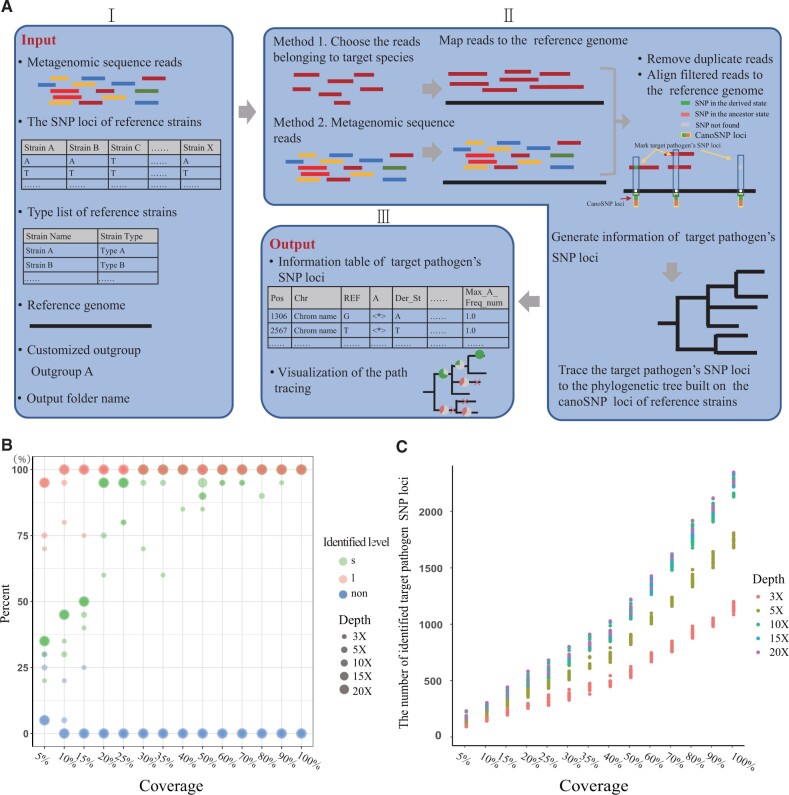
mStrain pipeline and the details of the identified *Y. pestis* in the 1400 *in silico* samples and six mouse blood samples. (A) The mStrain pipeline. (B) The number of in silico samples where *Y. pestis* was identified to the levels of strain (s), lineage (l), or not-found (non). The horizontal axis is the coverage of the selected genome to the total length of the *Y. pestis* chromosome genome. The coverage and sequencing depth are both calculated based on the reads belonging to the target pathogen *Y. pestis*. (C) The number of identified target pathogen SNP loci in the 1400 *in silico* samples.

The framework of mStrain contains two main steps: SNP identification and precise source tracking of target genomes (Fig. 1A). The details of each step to identify *Y. pestis* in metagenomic data are as follows.

For SNP identification of *Y. pestis* genome sequence in samples, mStrain uses two methods to extract reads belonging to *Y. pestis* (Fig. 1A II, Method 1 and Method 2). To exclude the reads from close relatives of *Y. pestis*, we used Kraken2 (v2.0.9) (Wood *et al.* 2019) to choose the reads belonging to *Y. pestis* before the alignment (Method 1). In Method 2, mStrain uses the BWA MEM (v0.7.17) aligner (Li 2013) to align the metagenomic raw reads to the *Y. pestis* reference genome CO92 and extracts aligned reads using SAMtools (v1.9) (Li *et al.* 2009). It is noted that mStrain aligns the reads to a single reference genome instead of a comprehensive dataset, which helps in reducing the computing time. Both Method 1 and Method 2 are capable of extracting reads that belong to *Y. pestis*, although they employ slightly different approaches to achieve this. We then use Picard (https://broadinstitute.github.io/picard/) to remove duplicate reads and Trimmomatic (v0.38) (Bolger *et al.* 2014) to remove reads shorter than 100 bp. Reads were transferred to a .bam file using the BWA-MEM (v0.7.17) aligner (Li 2013). We used BCFtools (v1.9) (Danecek *et al.* 2021) to filter SNP loci with base quality <30. We then compared filtered SNPs with the prebuilt *Y. pestis* canoSNP (Cui *et al.* 2013). CanoSNPs were generated using 134 strains, including 133 *Y. pestis* representative strains and the ancestor *Yersinia pseudotuberculosis* strain IP32953. We then removed SNPs with sequencing depths <3× and allele frequencies < 90%. Finally, we identified SNP loci of the *Y. pestis* strain in metagenomic data. These loci formed an information table with 13 attributes, including position (Pos), chromosome (Chr), reference (REF), allele (A), derived state (Der_St), ancestor state (Anc_St), node (Node), all depth (All_Dpt), filter depth (Fltr_Dpt), depth of the allele identical to the reference base (A_Ref_Dpt), depth of the derived allele (Der_A_St), heterozygote ratio (Het_Ri), variation type (Var_Type), and max-allele frequency number (Max_A_Freq_Num).

In the second step of precise source tracking, we first built a phylogenetic tree based on the canoSNPs using IQ-TREE (v1.6.5) (Nguyen *et al.* 2015). mStrain then traced back *Y. pestis* in the metagenomic data based on collected SNPs (Supplementary Table S1). Specifically, mStrain traverses SNPs in the phylogenetic tree structure and determines whether to stop traversing based on SNP state. We first input the root node (root_node) of the phylogenetic tree into the function get_loc and use the function child to obtain child nodes under the root node. We then traverse the child nodes and calculate the number of SNPs in the ancestor states. If the number is not equal to 0, we stop traversing the child node and mark the node on the phylogenetic tree with “X” to indicate that the genotype of *Y. pestis* may not belong to the branch where the node is located. Otherwise, we continue to traverse the sub-child nodes under the child node. If we can traverse the SNPs to the terminal node, then *Y. pestis* in the metagenomic data can be identified as the strain shown at the terminal node. Thus, we identify *Y. pestis* at the strain level. However, if we traverse the SNPs to a middle node, *Y. pestis* is considered to belong to the lineage where the middle node is located. We then used R software and related packages (Yu *et al.* 2017, 2018) to draw circular diagrams on each branch of the phylogenetic trees. The circular diagram displays the percentage of the three states of SNPs, including ancestor sate, derived state and not-found, with the number of SNPs of each type in the branch as a proportion of the total number of SNPs in the branch. By tracing back the SNPs using the phylogenetic tree (Supplementary Table S1), mStrain finally identified the target strain in the metagenomic data at the strain level (type s), lineage level (type l), and not-found (type non).

### 2.2 Datasets used in this study

To verify our mStrain method, we used three different datasets, including 1400 *in silico Y. pestis* metagenomic data, six mouse blood samples containing low concentrations of *Y. pestis*, and three clinical samples. Specifically, the 1400 *in silico Y. pestis* metagenomic data were generated by adding the simulated *Y. pestis* sequencing reads using ART (Huang *et al.* 2012) with different sequencing depths and coverages to the clinical sample sequencing reads. The six mouse blood samples were prepared by adding a concentration of 5 × 10^2^–5 × 10^4^ CFU/ml of *Y. pestis* to blood samples. Three clinical samples were obtained from the sputum and urine of two patients infected by *Y. pestis*. More details on the three datasets can be found in the Supplementary Material.

To fully assess mStrain for *Y. pestis*, we also included two negative control samples containing no *Y. pestis* strains. Specifically, we first employed a *Vibrio fluvialis* sample (HY41) as one negative control sample. To further generate the other *in silico* negative control sample involving off-target close relative *Y. pseudotuberculosis*, we enhanced the *V. fluvialis* sample’s sequencing reads by incorporating simulated sequencing reads from *Y. pseudotuberculosis* strain IP32953. This augmentation involved generating *Y. pseudotuberculosis* sequencing reads with a depth of 10× and ensuring 100% coverage.

## 3 Results

We initially validated mStrain using the two negative control samples without and with off-target close relative *Y. pseudotuberculosis*. In one negative control sample without *Y. pseudotuberculosis*, mStrain correctly detected the presence of the added *V. fluvialis* while not identifying any *Y. pestis* (Supplementary Fig. S3A). In the other negative control sample containing off-target close relative *Y. pseudotuberculosis*, mStrain accurately identified the presence of *Y. pseudotuberculosis* instead of *Y. pestis* (Supplementary Fig. S3B). These findings demonstrate that mStrain can successfully identify the intended target pathogens within the samples.

We used three different datasets to verify mStrain of *Y. pestis* detection, including *in silico Y. pestis* metagenomic data, blood samples containing low concentrations of *Y. pestis*, and clinical samples. mStrain identified *Y. pestis* at the strain level in each dataset. When processing a compressed FASTQ data sample size of 1.4 GB for the metagenomic sample, mStrain demonstrates a runtime of 4 min and consumes <60.0 GB of memory on a 32-core server. For a larger sample size of 235.0 GB of compressed FASTQ data, mStrain requires 4 h to complete and utilizes <50.0 GB of memory on the same 32-core server.

In the realm of lineage-level identification, mStrain accurately recognized *Y. pestis* in 1376 out of 1400 simulated metagenomic samples, achieving strain-level discrimination in 1158 of these instances (Fig. 1B, Supplementary Table S2). The number of *Y. pestis* SNPs identified was positively correlated with sequencing depth and coverage (Fig. 1C). In 90% of metagenomic samples, the lower threshold for identifying *Y. pestis* at the strain level fell within a range of (10× sequencing depth, 20% coverage) and (5× sequencing depth, 30% coverage) (Fig. 1B). The lower coverage may be compensated for with greater depth, and vice versa. In other words, if the sequencing depth is lower (e.g. 5× depth), then coverage should be higher (e.g. 30% coverage), and vice versa.

In mouse blood samples, mStrain accurately identified *Y. pestis* at the strain level in one and at the lineage level in two samples. Specifically, mStrain accurately identified the added *Y. pestis* strain EV76 to the strain level of node 1.ORI3a on the phylogenetic tree, using 191 SNPs, at a *Y. pestis* concentration of 5 × 10^4^ CFU/ml, 1× depth, and 15% coverage (Supplementary Fig. S1 M1). mStrain identified the added *Y. pestis* strain EV 76 to the lineage level 1.ORI using only three SNPs, at a *Y. pestis* concentration of 5 × 10^3^ CFU/ml, 1× depth, and 4% coverage (Supplementary Fig. S1 M2). Meanwhile, the lineage for the added *Y. pestis* strain 201 was identified as 0.PE4C, at a concentration of 5 × 10^4^ CFU/ml, 1× depth, and 12% coverage (Supplementary Fig. S1 M4).

In the clinical samples tested, mStrain successfully identified *Y. pestis* in one patient’s sputum to strain level 2.MED3m (Supplementary Fig. S2 and Table S3), with 1× depth and 38% coverage. Due to the early use of antibiotics, *Y. pestis* in these clinical samples could not be cultured using traditional methods. Therefore, we were not able to compare our results with whole-genome sequencing after culture. However, our result is consistent with previous Multiple Locus Variable-number tandem repeat Analysis (MLVA) results (Li *et al.* 2021), which identified the *Y. pestis* in this plague sample as 2.MED3m.

We evaluated the precision and recall of mStrain by analyzing the three different sample datasets (Supplementary Table S5). For *in silico* samples, mStrain achieves the precision of 100%/100% and recall of 98.3%/82.7% at the species/strain level. For wet-lab samples, mStrain achieves the precision of 100%/50% and recall of 50%/16.7% at the species/strain level. For clinical samples, mStrain has the precision of 100%/100% and recall of 66.7%/33.3% at the species/strain level.

The average sequencing depth of *Y. pestis* in wet-lab and clinical samples is typically 1×, below the lower 5× threshold for *in silico* samples. This low sequencing depth is likely the main reason why identifying *Y. pestis* in wet-lab and clinical samples is difficult. Despite the challenges posed by low concentration and sequencing depth, mStrain was still able to accurately identify *Y. pestis* in clinical samples, demonstrating its potential as an effective tool for source tracking of *Y. pestis* pathogens in metagenomic data at the strain level.

## 4 Conclusion

Strain-level target pathogen identification is essential for the prevention and control of infectious disease outbreaks. Owing to the low concentration of target pathogens in samples subjected to metagenomic analysis, strain-level identification is a great challenge. Herein, we propose mStrain as an efficient method for strain-level pathogen identification in metagenomic samples, which is based on the alignment between *Y. pestis* SNPs and canoSNPs. Our results showed that mStrain can accurately identify *Y. pestis* strains in metagenomic data and clinical samples. Taken together, mStrain can be used as a framework for strain-level target pathogen identification in metagenomic data.

## Supplementary Material

vbad115_Supplementary_Data
