# Supplementary material for: mStrain: strain-level identification of Yersinia pestis using metagenomic data
Source: Bioinform Adv. 2023 Sep 15;3(1):vbad115. doi: 10.1093/bioadv/vbad115 (PMC10516513; doi:10.1093/bioadv/vbad115)
Supplement: vbad115_Supplementary_Data [file vbad115_supplementary_data.docx]

Supplementary Material for “mStrain -- Strain-level Identification of Yersinia pestis Using Metagenomic Data”

By Xiuwei Qian, Yarong Wu, Xiujuan Zuo, Xin Peng, Yan Guo, Ruifu Yang, Xianglilan Zhang, Yujun Cui

Datasets used in this study

To verify our method mStrain, we used three different datasets, including *in-silico Y. pestis* metagenomic sequence data, blood samples with a low concentration of *Y. pestis*, and clinical samples.

We generated 1,400 *in-silico Y. pestis* metagenomic sequence data based on the representative whole genomes of *Y. pestis* and the high-throughput sequencing (HTS) reads of clinical samples. In particular, we randomly selected one *Y. pestis* strain from each of the five *Y. pestis* strain levels (2. ANT1c, 2.MED1a, 0.PE4Aa, 1.ORI3a, 0.PE4Cf, Table S4) and downloaded their whole genomes from the National Center for Biotechnology Information (NCBI) genome database. Next, we used ART (Huang *et al.*, 2012) to simulate synthetic next-generation sequencing reads generated by the sequencing system ID HS25 (HiSeq 2500, 150 bp), setting the sequencing depth, parameter f, as 3X, 5X, 10X, 15X, and 20X. We also wrote an in-house script to simulate the coverage level of 5%, 10%, 15%, 20%, 25%, 30%, 35%, 40%, 50%, 60%, 70%, 80%, 90%, 100%, separately. In order to mimic real metagenomic samples containing very low concentrations of *Y. pestis*, we added the simulated *Y. pestis* sequencing reads with different sequencing depths and coverages (from 1 M to 100 M data) to the real clinical samples that removed the plague sequences (~ 1.4 GB data/sample).

We then generated six biological samples by adding very low concentrations of *Y. pestis* to the blood samples. Specifically, we first diluted *Y. pestis* strains EV76 or 201 at different gradients (10^-3^, 10^-4^, 10^-5^) and then mixed them with other common pathogens (*Klebsiella pneumoniae, Yersinia pestis, Yersinia pseudotuberculosis*, etc.) at different concentrations in each blood sample (Figure. S1). These blood samples were then sequenced using the Illumina HiSeq platform. Finally, we acquired 150 bp HTS paired-end reads of six blood samples with very low concentrations of *Y. pestis* (~ 8.7 GB data/sample).

Three clinical samples were obtained from the sputum and urine of two patients, A and B, from Inner Mongolia, who were medically treated in Beijing in November 2019. DNA was extracted from these samples and then sequenced using the BGI MGISEQ-2000 sequencing platform. Finally, 2 × 150 bp paired-end reads were generated, and we acquired ~ 224.5 GB data per sample.

All procedures in this study were performed in accordance with the ethical standards of the National Research Council. All epidemiological investigations were performed in accordance with ethical standards, and verbal consent was obtained from each patient.

Supplementary tables and figure

Table S1. Pseudo code of tracing back the target pathogen using the phylogenetic tree built on the canoSNPs in mStrain

| function (get_loc (node)) |
| --- |
| 1: **Input**: root_node |
| 2: child nodes <- child (phylogenetic tree, root_node) |
| 3: **for** i in child nodes **do** |
| 4: a <- num (i (the SNP at ancestor state)) |
| 5: **if** a != 0 |
| 6: plot () |
| 7: **else**: get_loc (i) |

Table S2. Sequence depths and coverages of the *Y. pestis* reads in 1,400 *in-silico* metagenomic samples. The number in the cell shows the number of *in-silico* samples which have *Y. pestis* identified. The *Y. pestis* in these *in-silico* samples were identified to the levels of strain (s, in green), lineage (l, in red), or non-found (non, in grey). The metagenomic samples where *Y. pestis* was identified to the strain level are also counted to the samples where *Y. pestis* was identified to the lineage level.

| **Coverage**  **Sequence**  **Depth** | **5%** | **10%** | **15%** | **20%** | **25%** | **30%** | **35%** | **40%** | **50%** | **60%** | **70%** | **80%** | **90%** | **100%** | **Level Identified** |
| --- | --- | --- | --- | --- | --- | --- | --- | --- | --- | --- | --- | --- | --- | --- | --- |
| 3X | 4 | 7 | 8 | 12 | 16 | 14 | 12 | 15 | 17 | 19 | 19 | 20 | 19 | 20 | s |
| 5X | 6 | 6 | 10 | 15 | 16 | 19 | 19 | 20 | 18 | 19 | 19 | 18 | 20 | 20 | s |
| 10X | 7 | 9 | 10 | 19 | 19 | 20 | 20 | 20 | 19 | 20 | 20 | 20 | 20 | 20 | s |
| 15X | 7 | 9 | 10 | 19 | 19 | 20 | 20 | 20 | 20 | 20 | 20 | 20 | 20 | 20 | s |
| 20X | 7 | 9 | 10 | 19 | 19 | 20 | 20 | 20 | 20 | 20 | 20 | 20 | 20 | 20 | s |
| 3X | 14 | 16 | 15 | 20 | 20 | 20 | 20 | 20 | 20 | 20 | 20 | 20 | 20 | 20 | l |
| 5X | 15 | 19 | 20 | 20 | 20 | 20 | 20 | 20 | 20 | 20 | 20 | 20 | 20 | 20 | l |
| 10X | 19 | 20 | 20 | 20 | 20 | 20 | 20 | 20 | 20 | 20 | 20 | 20 | 20 | 20 | l |
| 15 | 19 | 20 | 20 | 20 | 20 | 20 | 20 | 20 | 20 | 20 | 20 | 20 | 20 | 20 | l |
| 20X | 19 | 20 | 20 | 20 | 20 | 20 | 20 | 20 | 20 | 20 | 20 | 20 | 20 | 20 | l |
| 3X | 6 | 4 | 5 | 0 | 0 | 0 | 0 | 0 | 0 | 0 | 0 | 0 | 0 | 0 | non |
| 5X | 5 | 1 | 0 | 0 | 0 | 0 | 0 | 0 | 0 | 0 | 0 | 0 | 0 | 0 | non |
| 10X | 1 | 0 | 0 | 0 | 0 | 0 | 0 | 0 | 0 | 0 | 0 | 0 | 0 | 0 | non |
| 15X | 1 | 0 | 0 | 0 | 0 | 0 | 0 | 0 | 0 | 0 | 0 | 0 | 0 | 0 | non |
| 20X | 1 | 0 | 0 | 0 | 0 | 0 | 0 | 0 | 0 | 0 | 0 | 0 | 0 | 0 | non |

Table S3. Details of mStrain-identified *Y. pestis* in the three clinical samples

| **Sample Name** | **Sample Source** | **Coverage of *Y. pestis* reads mapping to the reference genome (NC_003143.1)** | **Level Identified** |
| --- | --- | --- | --- |
| AT | Patient A’s sputum | 38% | s (2.MED3m) |
| BT | Patient B’s sputum | 15% | non (non-found) |
| BN | Patient B’s urine | 29% | non (non-found) |

Table S4. Basic information of the five strains used in the *in-silico* samples

| **Strain** | **Strain Level** | **Size (M)** | **Accession Number** | **Accession Site** |
| --- | --- | --- | --- | --- |
| 91001 | 0.PE4.Cf | 4.5 | NC_005810.1 | ftp://ftp.ncbi.nlm.nih.gov/genomes/all/GCF/000/007/885/GCF_000007885.1_ASM788v1 |
| KIM | 2.MED1a | 4.5 | NC_004088.1 | ftp://ftp.ncbi.nlm.nih.gov/genomes/all/GCF/000/006/645/GCF_000006645.1_ASM664v1 |
| 12 | 0.PE4.Aa | 4.37 | GCA_000320425.1 | ftp://ftp.ncbi.nlm.nih.gov/genomes/all/GCF/000/320/425/GCF_000320425.1_ASM32042v1 |
| EV76 | 1.ORI3a | 4.34 | GCA_000324805.2 | ftp://ftp.ncbi.nlm.nih.gov/genomes/all/GCF/000/324/805/GCF_000324805.2_EV76-CN |
| Nepal516 | 2.ANT1c | 4.4 | NC_008149.1 | ftp://ftp.ncbi.nlm.nih.gov/genomes/all/GCF/000/013/805/GCF_000013805.1_ASM1380v1 |

Table S5. The result of a benchmark test based on mStrain. The table contains precision, recall, memory, running time information.

| **Level Identified** | ***In-silico* Samples** | | | | **Wet-lab Samples** | | | | **Clinical Samples** | | | |
| --- | --- | --- | --- | --- | --- | --- | --- | --- | --- | --- | --- | --- |
|  | Precision | Recall | Memory | Running time | Precision | Recall | Memory | Running time | Precision | Recall | Memory | Running time |
| Strain Level | 100% | 82.7% | 60G | 4mins | 50% | 16.7% | 60G | 30mins | 100% | 33.3% | 50G | 4h |
| Species Level | 100% | 98.3% |  |  | 100% | 50% |  |  | 100% | 66.7% |  |  |

**
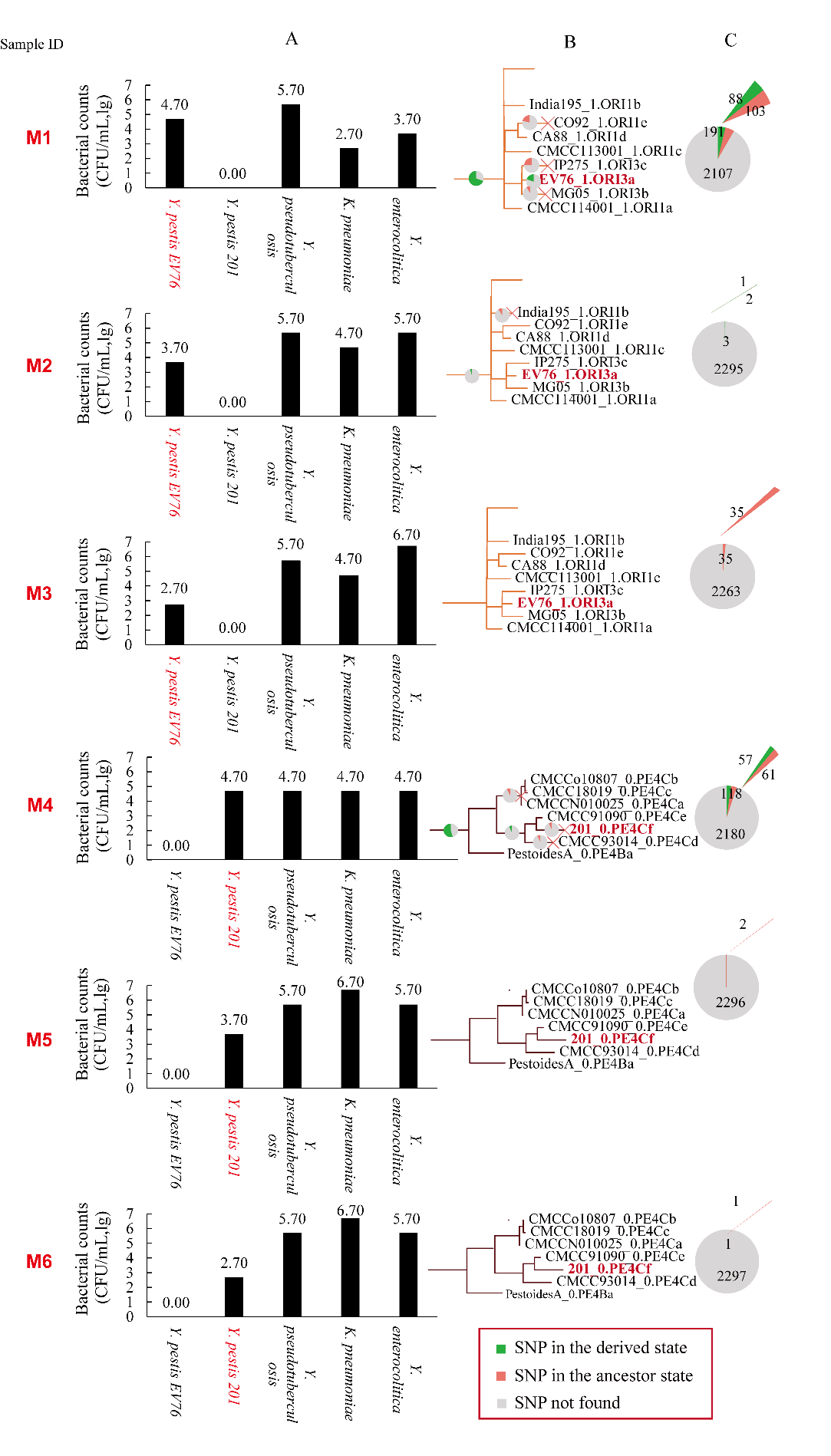
**

Figure S1 The identification of *Y. pestis* in the six blood samples. The target *Y. pestis* added in the mouse blood samples is highlighted in red in the concentration list and the phylogenetic tree that contains the target *Y. pestis*. The node name in the phylogenetic tree is displayed as “strain name_its strain type”

**
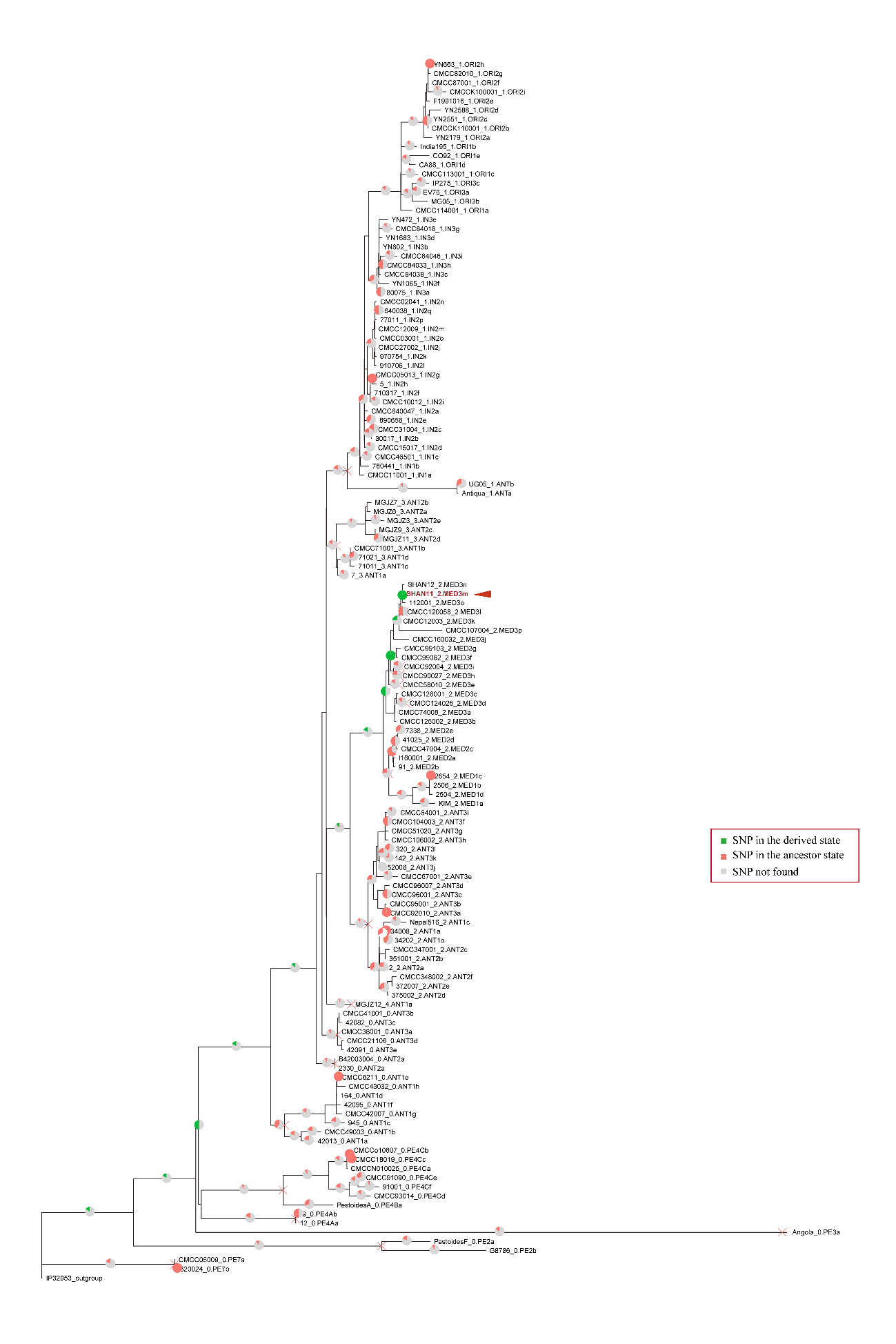
**

Figure S2. Details of tracing back strain-level *Y. pestis* in the clinical sample from patient A’s sputum. Pie chart in front of each node/branch represents the constitution of total SNP loci to identify this node/branch, where the SNP in the derived state is in green, the SNP at the ancestor state is in red, and the SNP not found is in grey. The ‘X’ in front of the branch represents that the *Y. pestis* in the metagenomic sample does not belong to this branch. The arrow illustrates the mStrain-identified strain level *Y. pestis*, strain-level 2.MED3m, in patient A’s sputum.
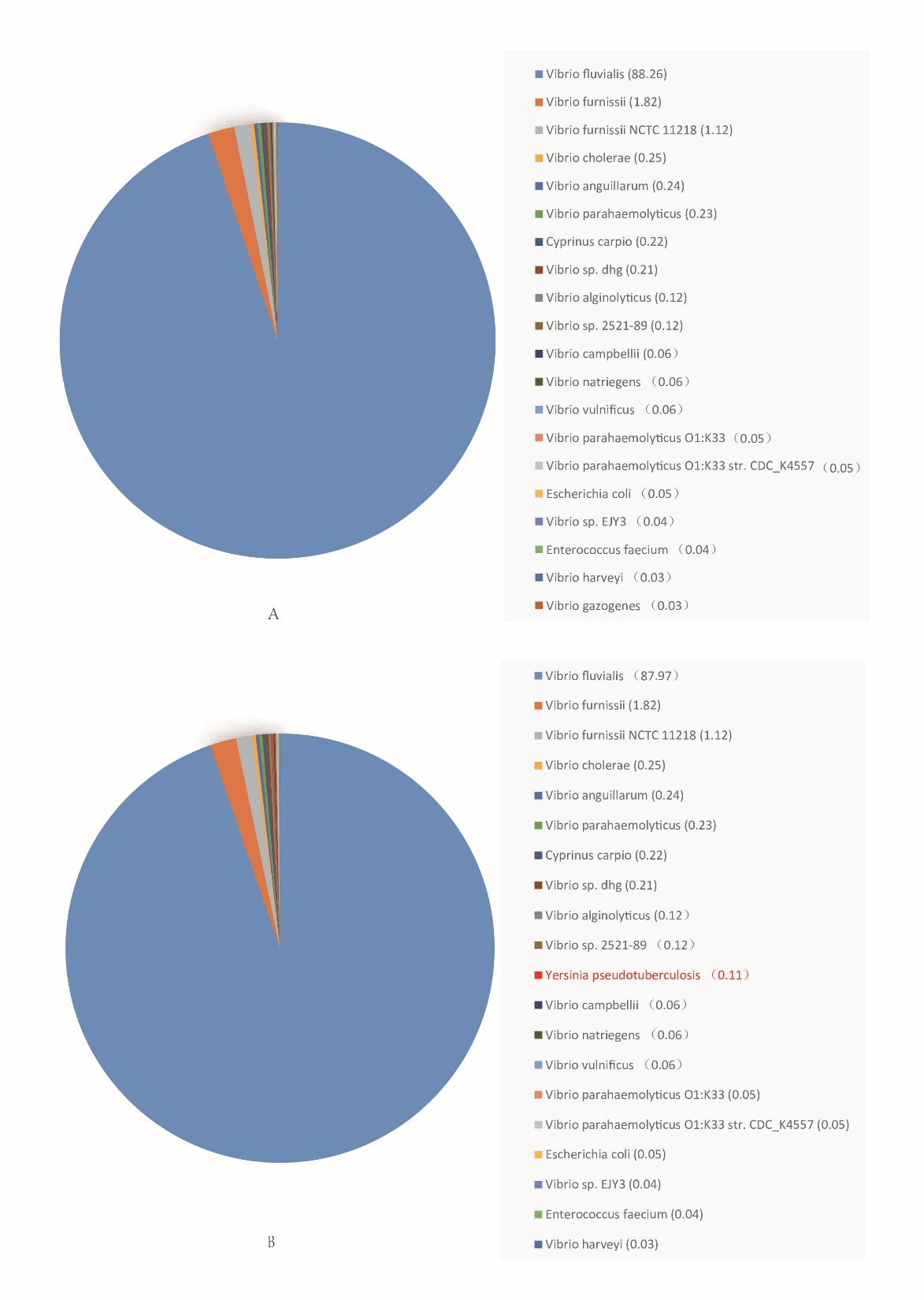


Figure S3. (A) A *Vibrio fluvialis* sample (HY41) as the negative control. (B) An *in-silico* *Y. pseudotuberculosis* metagenomic data by adding the simulated *Y. pseudotuberculosis* sequencing reads with sequencing depth of 10X and coverage of 100% to the *V. fluvialis* sample sequencing reads. mStrain accurately identified the presence of *Y. pseudotuberculosis* instead of *Y. pestis*.
